# Synchrony 2022: The Role of Neuroinflammation in Behavioral Exacerbations in Autism Spectrum Disorder

**DOI:** 10.3390/jpm13071133

**Published:** 2023-07-13

**Authors:** Jennifer Frankovich, Heer Nanda, Elizabeth D. Mellins, Harumi Jyonouchi, Richard G. Boles, Stephen J. Walker, John Gaitanis, Richard E. Frye

**Affiliations:** 1 Lucile Packard Children’s Hospital, Stanford School of Medicine, Stanford, CA 94010, USA; jfranko@stanford.edu (J.F.); mellins@stanford.edu (E.D.M.); 2 University of California, Berkeley, CA 94720, USA; heer.nanda@berkeley.edu; 3 St. Peter’s University Hospital, New Brunswick, NJ 08901, USA; hjyonouchi@saintpetersuh.com; 4 NeurAbilities, Vorhees, NJ 08043, USA; rboles@neurabilities.com; 5 NeuroNeeds^®^, Old Lyme, CT 06371, USA; 6 Wake Forest Institute for Regenerative Medicine, Winston Salem, NC 27101, USA; swalker@wakehealth.edu; 7 Hasbro Children’s Hospital, Brown University, Providence, RI 02903, USA; jgaitanis@lifespan.org; 8 Autism Discovery and Treatment Foundation, Phoenix, AZ 85050, USA

The BRAIN Foundation (Pleasanton, CA) hosted Synchrony 2022, a medical conference focusing on research for treatments to benefit individuals with neurodevelopmental disorders (NDD), including those with autism spectrum disorders (ASD). One of the four roundtables focusing on some of the most challenging and unsolved problems in ASD concentrated on understanding behavioral exacerbation in children with ASD and gaining a better understanding of the underlying causes. 

Dr. Jennifer Frankovich, MD, MS Director of the Immune Behavioral Health Clinic and Clinical Professor in the Dept of Pediatrics at Stanford Children’s Health/Lucile Packard Children’s Hospital chaired a roundtable with experts in behavior exacerbation in children with ASD and the possible role of systemic or localized inflammation in such behavioral exacerbations. While selected experts provided specific input, significant time was also devoted to discussing the important knowledge gaps, input from parents regarding unmet needs, and case presentations from physicians and parents to illustrate some of the most difficult cases.

Additional panelists included Dr. Richard E Frye, MD, PhD, President of the Autism Discovery and Treatment Foundation; Elizabeth D. Mellins, MD from Stanford University; Harumi Jyonouchi, MD from St. Peter’s University Hospital; Stephen J. Walker, PhD from the Institute for Regenerative Medicine at Wake Forest; John Gaitanis, Chief of Child Neurology at Hasbro Children’s Hospital, Brown University; and James Adams, Director of the Autism Program at Arizona State University.

The panel’s first task was to consider the overlap of ASD with Pediatric Acute-onset Neuropsychiatric Syndrome (PANS) and Pediatric Acute-onset Neuropsychiatric Disorder associated with Streptococcus (PANDAS). The panel recognized that the symptoms overlapped between ASD and PANS/PANDAS. However, one of the concerns from a diagnostic perspective was that, in PANS/PANDAS, the defining symptoms can arise suddenly in individuals with either low-grade symptoms or without any preceding symptomatology, in contrast to individuals with ASD, where symptomatology may worsen rather than arise “out of the blue”. One substantial concern from a diagnostic perspective regards the threshold for how much worsening of symptoms is considered a significant exacerbation versus a de novo manifestation. In addition, the emergence of neuropsychiatric symptoms as sequelae of the coronavirus disease of 2019 (COVID-19), which is now referred to as long COVID, may also cause changes in behavioral symptoms in some ASD subjects. 

Parents in attendance remarked about repeated relapses and remissions of neuropsychiatric exacerbation throughout childhood, adolescence, and adulthood, which were associated with infections and onset/relapses of other comorbidities (inflammatory bowel disease, psoriasis, arthritis, epilepsy, etc.) and possibly dietary and other environmental triggers. 

Thus, in an effort to move forward, the panelists agreed to work together to develop a comprehensive consensus paper that outlines the current knowledge regarding behavioral exacerbation in ASD. An additional aim is to identify research approaches to elucidate underlying causes and ultimately to develop an evidence-based differential diagnosis instrument. Initially, the process will be developed based on a review of the literature and expert opinion on the important underlying causes of behavioral exacerbations. However, in the longer term, this diagnostic tool will need to be validated, perhaps through the identification of noninvasive biomarkers associated with the various causes of exacerbations, allowing targeted treatments. Overall, the consensus was that this is an important area of ASD research, which currently has many knowledge gaps. Further, the group felt that better defining the phenomenon of behavioral exacerbation in ASD and its underlying causes could significantly improve the quality of life of children and adults with ASD and their families. 

